# The Most Recent Advances in the Application of Nano-Structures/Nano-Materials for Single-Cell Sampling

**DOI:** 10.3389/fchem.2020.00718

**Published:** 2020-08-20

**Authors:** Xiaolong Xu, Jianbo Jia, Mingquan Guo

**Affiliations:** ^1^School of Biotechnology and Health Science, Wuyi University, Jiangmen, China; ^2^CAS Key Laboratory of Plant Germplasm Enhancement and Specialty Agriculture, Chinese Academy of Sciences, Wuhan, China

**Keywords:** single-cell sampling, nanostructure, nanopipette, nanostraw, carbon nanotube probes

## Abstract

The research in endogenous biomolecules from a single cell has grown rapidly in recent years since it is critical for dissecting and scrutinizing the complexity of heterogeneous tissues, especially under pathological conditions, and it is also of key importance to understand the biological processes and cellular responses to various perturbations without the limitation of population averaging. Although conventional techniques, such as micromanipulation or cell sorting methods, are already used along with subsequent molecular examinations, it remains a big challenge to develop new approaches to manipulate and directly extract small quantities of cytosol from single living cells. In this sense, nanostructure or nanomaterial may play a critical role in overcoming these challenges in cellular manipulation and extraction of very small quantities of cells, and provide a powerful alternative to conventional techniques. Since the nanostructures or nanomaterial could build channels between intracellular and extracellular components across cell membrane, through which cytosol could be pumped out and transferred to downstream analyses. In this review, we will first brief the traditional methods for single cell analyses, and then shift our focus to some most promising methods for single-cell sampling with nanostructures, such as glass nanopipette, nanostraw, carbon nanotube probes and other nanomaterial. In this context, particular attentions will be paid to their principles, preparations, operations, superiorities and drawbacks, and meanwhile the great potential of nano-materials for single-cell sampling will also be highlighted and prospected.

## Introduction

The presence of heterogeneity in cell populations calls for inspection down to single-cell level in nearly all fields of biology and medicine. The unique features of individual cells, even originally with the same genetic information, and from the same multicellular organisms, include but not limited to their structure, composition, and functionality (Guillaume-Gentil et al., 2016). It has been reported an unexpected level of somatic genomic variations in both normal and diseased tissues (Gupta and Sachs, 2017). Taking acute myeloid leukemia (AML) for example, it is a heterogeneous disease both at phenotypic and genotypic levels, and this heterogeneity extends to leukemia stem cells (Gupta and Sachs, 2017). Differential gene expression leads to the diversity of cell phenotypes resulting in individual cells with highly specialized functions. On the other hand, the randomness of intracellular processes, together with changes in the environment around, further gives rise to different cellular functions, even in the homogenous cell populations. Thus, molecular analysis at a single cell level is essential for the evaluation and inspection of the complexity of heterogeneous tissue, the description of pathological conditions, the study of biological processes, and the cellular response to disturbances, without masking cellular heterogeneity (Guillaume-Gentil et al., 2016). To this end, it is crucial to explore where and when biomolecules exert their functions in regulating the activity of cells. In order to know what is going on in the life cycle of a cell, it is fundamental to elucidate the molecular composition at a given site in real time. These efforts will of key importance not only to understand cell activity and its controlled differentiation, but also to realize the potentially targeted therapy of living cells.

It is well known that the spatial resolution of conventional optical microscopy can reach about 200–300 nm due to the Abbe diffraction limit, which is unable to visualize most of the biomolecule individually in a single living cell. To improve the spatial resolution, the supper resolution optical microscopies have been actively developed, such as stimulated emission microscopy (STED), photoactivated localization microscopy (PALM), and stochastic optical reconstruction microscopy (STORM). Their spatial resolution could achieve down to several ten nanometers, and fluorescence labeling is also necessary. In most cases, the fluorescence image is taken from fluorescent labeling reagent itself instead of the target molecules. Besides, fluorescence labeling may affect the behaviors of cells. To overcome the above limitations, transmission electron microscopy (TEM) and scanning electron microscopy (SEM) have been developed with superior spatial resolution, which can visualize single molecules, but cannot be carried out directly on living cells. Although powerful, these methods are hampered by cell lysis in advance to extract the intracellular contents, which can provide only a single instant snapshot without historical or future information about the cell cycle. Thus, how to conduct accurate sampling from a single cell becomes the key for single cell analysis.

Successful techniques for sampling from a single cell, in other words, extracting small quantities from one or multiple sites into a single cell for long-term tracking of interested activity, must be able to manipulate picoliter-scale volumes with high cell viability, and to accurately reflect the cell's multiple biological components but without influencing the ongoing development of the cell (Higgins and Stevens, 2017). In this sense, nanostructure or nanomaterial may play a critical role in overcoming these challenges, since the nanostructures or nanomaterial could build channels between intracellular and extracellular components across cell membrane, through which cytosol could be pumped out and transferred to downstream analyses. Some recent reviews discussed these questions from different angles. Traditional single cell analysis calls for sampling inside single cell, like RNA or DNA (Sharma et al., 2018). So far, electroporation is still one of the first choices to transport biomolecules across the membrane into or out of the cell (Napotnik and Miklavcic, 2018). Kim and Lee have reported on the delivery of nanoparticles as intracellular carriers by electroporation (Kim and Lee, 2017). Tay and Melosh have tried the tubular nanostructures for cargo delivery into cells (Tay and Melosh, 2019). More recently, nanopipette has shown great potential in DNA detection *in vitro*, it also demonstrates the ability to sample from nucleoplasm or cytoplasm (Wang et al., 2019). Meanwhile, applications of AFM and FluidFM technologies in molecular and cellular biology have also been reviewed recently, especially in the aspects, such as cellular morphology, cellular mechanics, and manipulation of a single cell (Amarouch et al., 2018; Li et al., 2019). In this review, we focus on some most promising methods for single-cell sampling with nanostructures, such as glass nanopipette, nanostraw, carbon nanotube probes and other nanomaterial. In this context, particular attentions will be paid to their principles, preparations, operations, superiorities and drawbacks, and meanwhile the great potential of nano-materials for single-cell sampling will also be highlighted and prospected.

## Single-Cell Sampling With Nanostructures

### Nanostraw for Single-Cell Sampling

**Figure 1:**
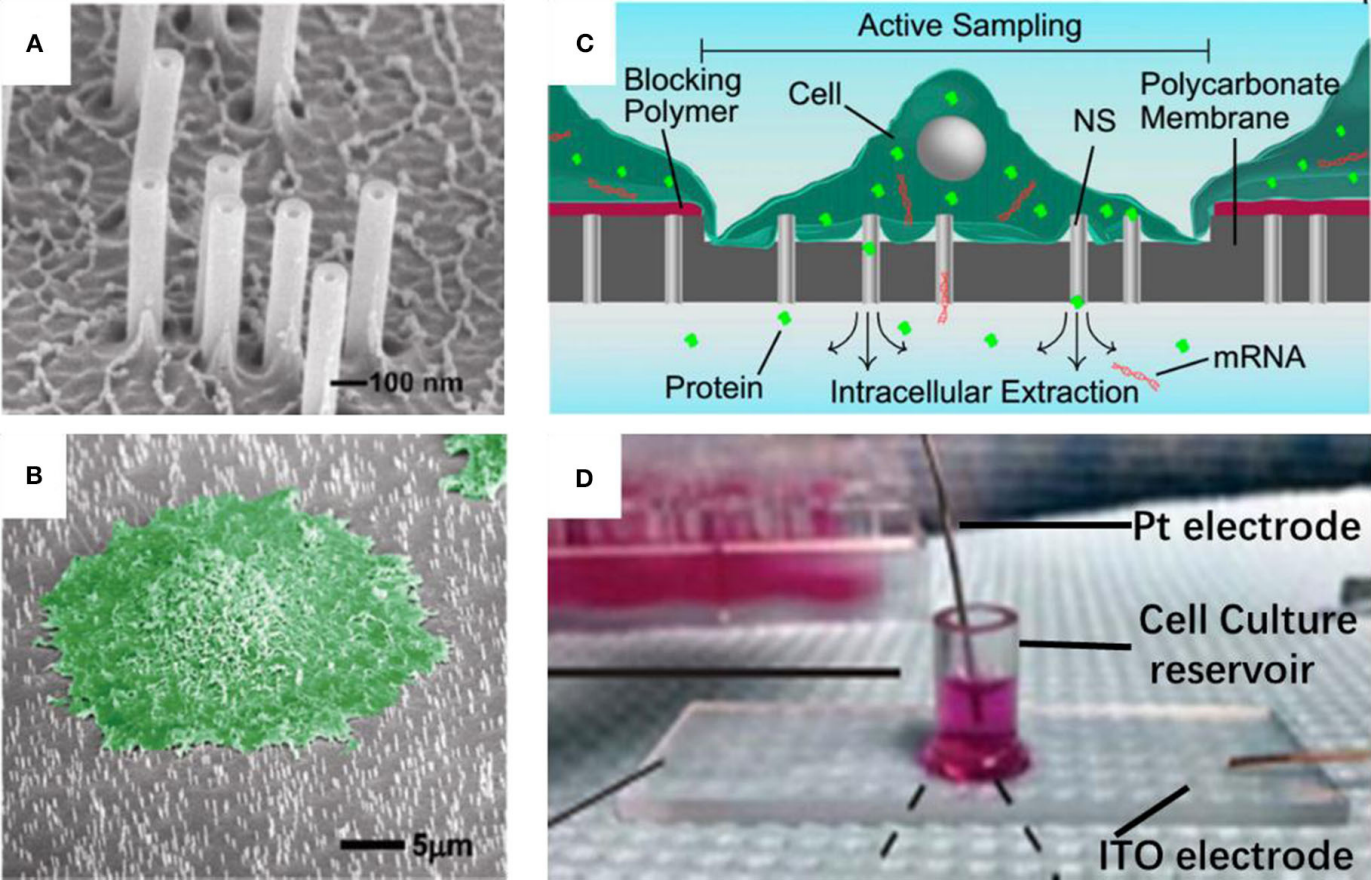
Scanning electron micrograph for nanostraw membrane **(A)** and critical point dried cell cultured on nanostraw membrane (**B**, false colored green); schematic **(C)** and device pictures **(D)** for nanostraw electroporation system.

Nanostraws, a random arrangement of hollow cylinders, were made from a variety of materials, such as alumina (VanDersarl et al., 2012; Cao et al., 2017, 2018; He et al., 2018), silica (Peer et al., 2012), silicon nitride (Huang et al., 2019). Typically, the alumina nanostraws were fabricated using track-etched polycarbonate membranes as the template, which are commercially available with different pore sizes and pore densities. Onto the template, an alumina coating was a uniform coating with atomic layer deposition (ALD), and typically 10–30 nm thick. The deposited alumina forms the nanostraw bodies with the nanopore interiors defining the nanostraw wall thickness. After removing the alumina on the top surface and part of the exposed polymer with reactive ion etching (RIE), the nanostraws were obtained, and then they could be put on top of a microfluidic channel, with cell loaded on the other side. In this way, a number of nanostraws covered by cells would penetrate through cell membranes steadily over extended periods. Thus, molecules in extracellular environment, such as ions or plasmids could diffuse from the microchannel into cytosol. In addition, the dimensions of a nanostraw, such as straw wall thickness, and nanostraw height could be independently tuned through adjustments to the track-etched membrane properties (straw outer diameter and density), as shown in Figure 1A (reprinted from VanDersarl et al., 2012). Amazingly, the highly uniform nanostraw has been reported with <5% variations for wall thickness, height, and inner diameters as measured by scanning electron microscope images. The authors found that the dimension was important for this method. Nanostraws with diameter of 100 nm could penetrate cell membranes, while larger ones (250 and 500 nm) couldn't. Also, the cell membrane penetration is a stochastic process with roughly <10% efficiency per nanostraw. Thus, it is very difficult to achieve an ideal straw density, since there must be a compromise between two competing effects. On the one hand, the lower straw concentrations may lead to lower total molecular flux through the membrane; on the other side, very high nanostraw densities could result in less frequent cell penetration, because there exists a bed-of-nails effect when cells resting on the top of the dense nanostraw forest, which is shown in Figure 1B (reprinted from VanDersarl et al., 2012).

As mentioned above, the connection between nanostraws and cytoplasm remains open after the penetration over extended periods. Although, such stable fluidic interfaces can facilitate temporal control of their delivery, it is also at risk of leakage of proteins and ions through these channels. Melosh's group developed an electroporation system with similar nanostraw array of large diameter (250 nm), which could not penetrate cell membrane itself. Thus, the fluidic interfaces are temporarily established once the voltage applied, followed by self-healing after removal of the voltage (Xie et al., 2013), as shown in Figures 1C,D (reprinted from Cao et al., 2017, 2018, respectively). Biomolecule delivery could be achieved by diffusion via the nanostraws and improved by electrophoresis during pulsing. This system could not only offer excellent spatial, temporal, and dose control for the biomolecule delivery, but also provide high-yield co-transfection and sequential transfection efficiency (Xie et al., 2013). In principle, substance exchange goes both directions once the fluidic interfaces established. From another point of view, the leakage of cytosolic contents could also be regarded as sampling (Cao et al., 2017; He et al., 2018). Melosh's group reported the nanostraws were used as time-resolved, longitudinal extraction method for intracellular proteins and mRNA. In a typical extraction process using this so-called nanostraw extraction system, approximately 5–10% of both big molecules, like proteins and mRNA, and small molecules could diffuse from cell passively, through the nanostraws, and into the extraction solution in microfluidic channels, which is on the other side of the nanostraws. In this way, repeat extractions from the same cell or cell population were demonstrated, and conventional methods, such as fluorescence, enzymatic assays (ELISA), and quantitative real-time PCR, could be used to analyze the extracted contents (Cao et al., 2017). The spatial resolution of the nanostraw extraction system was realized by microfluidic channel design, yet subcellular resolution was not available since the sample spots, and nanostraws-cell interfaces were at a stochastic distribution. It has been estimated that underneath a typically 10 μm × 10 μm adherent cell could be tens to hundreds of nanostraws with only a small portion of them communicating with cytosol stochastically (VanDersarl et al., 2012; Cao et al., 2017). Meanwhile, direct sample extraction from cargo delivery to cell nucleus are still challenged.

In a recent report, the gold coated nanostraws were reported for on-demand intercellular delivery of single particles into a single cell, and they were shaped on a Si_3_N_4_ substrate which was embedded in a polydimethylsiloxane (PDMS) chamber. The gold coated nanostraws acted simultaneously as nanoelectrodes for electroporation and fluidic interface for delivery of nanoparticles, and also as plasmonic antennas for the enhancement of Raman signals. When illuminated with laser, the gold coated nanostraws were capable of confinement and enhancement of electromagnetic fields, and able to discern the SERS signals from a single nanoparticle upon flowing through the nanostraws. By this means, the delivery of single nanoparticle into a selected cell was accurately counted by SERS (Huang et al., 2019).

### Nanoneedle for Single-Cell Sampling

Atomic force microscopy (AFM), a very powerful tool for surface image, and it can be used to scan the sample by a pyramidal tip on a flexible cantilever spring. While the tip is scanning over the sample surface, the interaction forces between the tip and the sample surface distort the cantilever. The distortion is monitored with a laser beam, and could be deduced into topographic image vs. relative position of the tip (Amarouch et al., 2018). Cargo, such as plasmid DNA or dyes, could be deliver into cell by loading them onto normal (Cuerrier et al., 2007) or sharpened AFM tips before its penetration through cell membrane (Obataya et al., 2005; Silberberg et al., 2013). This sharpened tip has more advantages, not only can it access the cytosol, but also penetrate through the nuclear membrane without chromosomal DNA damage or apoptosis, once it is effectively inserted through the plasma membrane of a living cell (Ryu et al., 2013). Moreover, sharpened AFM tips modified with specific antibodies could be used to assess the unbinding forces during evacuation of the tip from the cell. Thus, specific mechanical interactions between the antibody-functionalized tip and the intracellular components could be measured and used for cell screen (Silberberg et al., 2013, 2014; Li et al., 2017, 2019).

AFM has also been used for imaging cell surfaces, estimating cell membrane properties, such as elasticity and viscosity. When decorated with plasmid DNA encoding for the fluorescent protein EGFP, the AFM tip could penetrate through the cell membrane and delivery the plasmid DNA into cell (Cuerrier et al., 2007). Ultrathin probes, such as modified AFM tips, could be developed as tools for single cell biopsy at nanoscale resolution in conjugation with AFM (Han et al., 2005). In this system, the nanoneedles were fabricated from AFM tips using focused ion beam (FIB) etching, with a diameter of 200 nm and a length of 6–8 μm. A molecular force probe was used for the manipulation and force measurement of the nanoneedle (Obataya et al., 2005). When inserting the nanoneedle into the cell, the preloaded plasmid DNA could be detached from the needle surface in about 5 min. This technique has advantages regarding accurate force feedback, which is helpful to judge the critical timing of the cell membrane puncture. Due to the small size and the high aspect ratio, the nanoneedle is a potent tool for the single cell inspection. For example, when antibody-immobilized nanoneedle was inserted into living cells, specific intracellular cytoskeletal proteins could be probed. While the inserting nanoneedle being retracted, the mechanical force to release the binding complexes between the antibody and target proteins could be measured, and in this way the intermediate filament protein, neurofilament and nestin in mouse embryonic carcinoma P19 cells or rat primary hippocampal cells were successfully detected (Mieda et al., 2012; Silberberg et al., 2013). In these cases, the penetration into the nucleus affect neither the doubling time of the cells nor double-stranded DNA (Ryu et al., 2013).

By a similar strategy, antibody-functionalized nanoneedle array was fabricated to target individual cells, and separate them from a mixed population of cells on a substrate (Kawamura et al., 2017). The nanoneedle array comprised of 10,000 nanoneedles was fabricated using top-down MEMS (Micro-Electro-Mechanical System) technique with each nanoneedle <200 nm diameter and more than 20 μm long. As a proof-of-concept demonstration, nanoneedles were modified with antibody. When this nanoneedle array was inserting into and retracting from the substrate adhesion cells mixture, target cells would be lifted out from the cell mixture due to the specific interactions between the nanoneedle and specific intracellular proteins inside the target cells. Although separation efficiency should be improved before practical applications, this approach is compatible with intact living cells. In the cell separation process, it does not need to transform the cells for fluorescent visualization of target protein expression as required by conventional methods, thus it does not need to remove fluorescently labeled antibodies either, since the antibodies are already covalently attached to the nanoneedle for the intracellular marker proteins. In this way, Yang and co-workers developed a method for the evaluation of enzyme activity using a live cell sandwich method with live cells between two silicon nanoneedle arrays. The substrate nanoneedle array was used to immobilized the cells, and the second nanoneedle array was covalently modified with enzymatic substrates. When the arrays were penetrated into cell membrane, these substrates interact with cytoplasmic enzymes, and the changes were monitored by conventional methods, such as fluorescence microscopy and mass spectrometry (Na et al., 2013).

**Figure 2:**
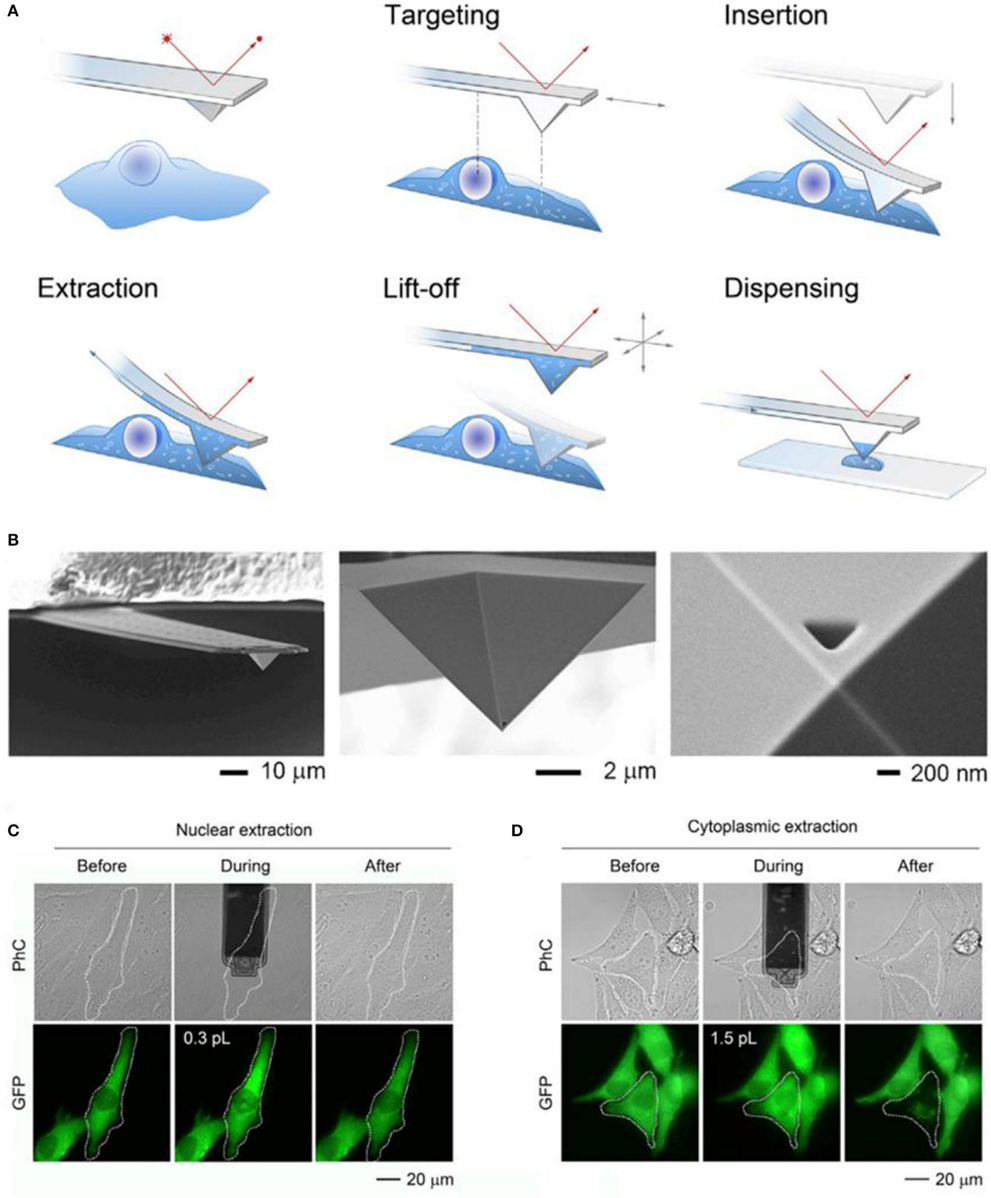
Schematic of the FluidFM based extraction procedure **(A)** and SEM image of a FluidFM probe **(B)**; phase-contrast (PhC) and fluorescent images with GFP of representative extracts from the nucleus **(C)** and cytoplasm **(D)** of the target cells.

Fluidic force microscopy (FluidFM), is another powerful tools for the single cell sampling, which combines a conventional AFM with microchannel cantilevers connected to a pressure controlled fluidic circuit, and able to manipulate liquid locally (Guillaume-Gentil et al., 2014; Amarouch et al., 2018). Quantitative extraction of samples from single cells with subcellular spatiotemporal control was demonstrated using FluidFM, and meanwhile, the soluble molecules withdrawn from the cytoplasm or nucleus could be analyzed by transferring the extract sample fluid to TEM, enzyme activity assays, and qPCR (Guillaume-Gentil et al., 2014; Amarouch et al., 2018). The activities of the extract samples were monitored with microscopy, and the volumes were calculated from the size of occupied microchannels with typical volumes ranging from 0.1 to 7.0 in a single cell, as shown in Figure 2 (reprinted from Guillaume-Gentil et al., 2016).

The aperture of the FluidFM probe is in the range of several tens to a few hundred nanometers. After shrinking the pore size down to a few nanometers provides extra function, the nanopore has been widely used for the single molecule detection and ion current recording. This was done by construction of a nanopore with diameter as small as 5 ± 1 nm onto the flattened apex of FluidFM probe, so called force-controlled scanning nanopore microscope. By this means, an integration of solid-state nanopore and AFM was accomplished, and it can be used to stochastically sense the secreted molecules, and the activity of ion channels in arbitrary locations both inside and outside a cell (Aramesh et al., 2019). Besides surface imaging, the detection and delivery of biomolecules on-demand, the introduced system also facilitates flexibility and controllable mechanical engagement with the target samples. In this sense, the translocation of biomolecules and ions through the nanopore can be observed in living cells and in real time, although some of the translocation signals could not be assigned to specific events, since the pristine nanopore detected signals are non-specific without further modification of the nanopore or construction of detectable specific interactions between target molecules and tags.

### Nanopipette for Single-Cell Sampling

Nanopipettes, with a conical shape and submicron to nanoscale size of the pore opening at the tip, are suitable for delivery of biomolecules to and/or from a single living cell, or as a probe for the cells. Nanopipettes are usually made from glass capillary by heating to soften the middle part, and then pulling it apart into twin nanopipettes. The most prevalent equipment is laser-based micropipette puller (P-2000, Sutter Instrument). Typically, a glass capillary loaded into the puller bar is laser-soften to a certain degree before hard pull, and the capillary is then separated into twin glass nanopipettes. This machine has five parameters to control the heat, pull, and timing in order to adjust the shape of the as-prepared nanopipettes: HEAT, the output power of the laser, and consequently the amount of energy supplied to the glass capillary; FILAMENT, the scanning pattern of the laser beam that is used to supply HEAT to the glass capillary; VELOCITY, the velocity at which the puller bar must be moving before the hard pull is executed; DELAY, the timing of the start of the hard pull relative to the deactivation of the laser; and PULL, the force of the hard pull. Although there are slight differences between instruments, nanopipettes with diameters of 10–300 nm can be successfully prepared by synergistically adjusting these five parameters. In terms of material selection, borosilicate glass or quartz are usually used to prepare nanopipettes, since quartz is capable of producing stronger and smaller tips, and borosilicate glass is easier to control and cheaper.

Structurally, the cavity of the nanopipettes could act as passage, through which many biological molecules, such as DNA and proteins, could be pressure or electrophoresis driven in and out of single cell cytosol. Conventional methods of cell injection employ micropipettes with tip diameters of 0.5–5 μm that is incompatible to puncture most cells. While nanopipette has a typical tip size <200 nm, there are several advantages, such as little disruption to the cell membrane structure and function, and ease to control the amount of interested substances. As for the driving force of the substance in and out of the cell, concentration gradient, potential difference, electroosmotic flow, electrophoresis and electrowetting are potential options in principle. Pressure, for most of the cases, is out of service for such small orifice, since the pressure applied to drive the substance is beyond the mechanical strength of the glass tip, result in the nanopipette to crush.

**Figure 3:**
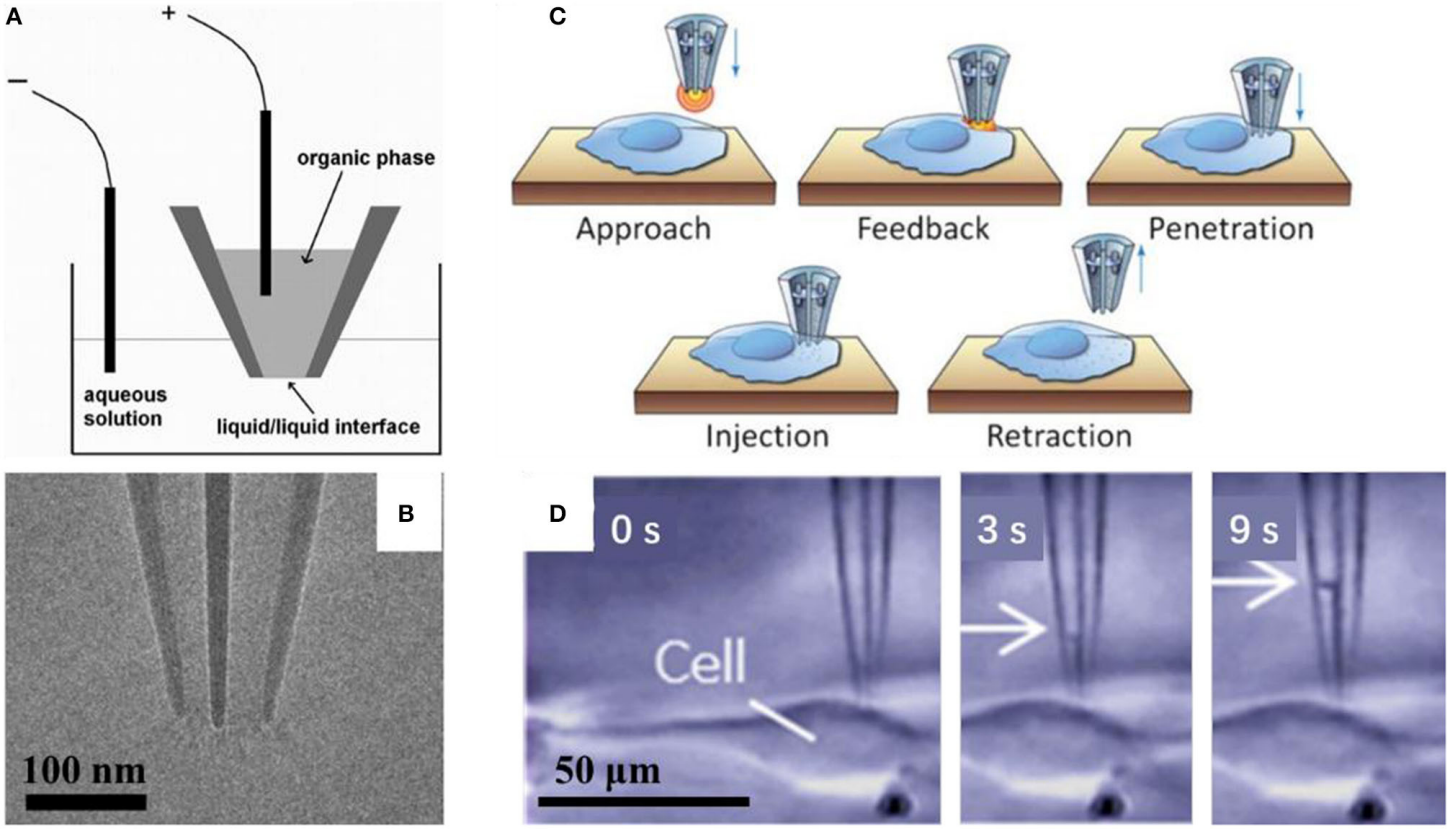
Scheme of the electrochemical attosyringe **(A)**; TEM image **(B)** and illustration of cell surface detection, penetration and injection **(C)**, and sequential micrographs acquired during cytosol collection **(D)** using a double-barrel nanopipette.

Mirkin's group had demonstrated that it was possible to control fluid motion electrochemically using nanopipette, as shown in Figure 3A (reprinted from Laforge et al., 2007). They filled the nanopipette with 1, 2-dichloroethane (DCE) and dipped it into aqueous solution, with one reference electrode inside the nanopipette and the other one in the aqueous solution. The potential difference between the two liquid phases could be controlled by applied voltage between these two electrodes, and then, the voltage may change the surface tension through the liquid/liquid interface, and in turn induce the corresponding force to evoke the liquid flow into/out of the nanopipette. When the negative potential is applied to the inner (organic) solution, the shape of the meniscus will change at the interface of the two liquids, and then cause water to enter the pipette. On the contrary, when a sufficiently positive potential is applied to the inner reference electrode, it will induce the expulsion of water (Laforge et al., 2007). With this tool, so called electrochemical atto-syringe, some dyes can be delivered, while they cannot cross a mammalian cell membrane, into a single cell. Volumes that can be manipulated may depend strongly on the orifice radius, and the duration and amplitude of the potential applied. The amount of liquid manipulated could be estimated by calculating the volume of the filled part of the nanopipette, or the corresponding current-voltage curves. At this stage, injection position distinction between cytoplasm and nucleus had not been reported. Extraction cytosol fluid from multiple locations in the same cell could be realized for mapping the various mRNA species to specific subcellular location (Toth et al., 2018).

A multiwalled carbon nanotube, with length of about 50–60 μm and 50 to more than 200 nm outer diameter, was fixed at the tip of micropipette as carbon-nanotube-based endoscope for interrogating the single cell (Singhal et al., 2011). In this case, the intracellular environments even organelles can be probed by the endoscope, and achieve a spatial resolution of about 100 nm without disrupting the cell. When magnetic nanoparticles are used to fill the nanotube, nanoparticles and atto-liter volumes of fluids can be remotely transferred to and from precise locations through the endoscope.

Scanning ion conductance microscopy (SICM), another scanning probe, has gained increasing attention, and can be used to image surfaces of living cells with a high spatial and temporal resolution. An electrolyte filled nanopipette could be used as the probe of SICM (Bulbul et al., 2018). When voltage bias applied, ions flow through the nanopipette orifice. As the nanopipette is scanning over a surface, the magnitude of the ion current may change which reflects the topography of the sample. In this sense, nanopipette could be used as a navigation probe close to target cell and fluidic interface for delivery or extraction of molecules interested into or from target cell. Pourmand's group achieved this by continuous sampling of intercellular contents from single cell (Actis et al., 2014). Subcellular resolution of sample was realized for the isolation of small subpopulations of mitochondria from single living cells. In this way, mutant mitochondrial genomes in those samples could be quantified with high throughput sequencing technology.

One of these reference electrodes could be integrated by sputter coating a thin layer of Ir/Pt outside the nanopipette (Shekaramiz et al., 2016, 2018). In this case, the whole system has a compact size with no influence on its performances. Shekaramiz and co-workers used this system to transfect single cells. The nanopipette had a tip opening about 140 nm, filled with 1, 2-dichloroethane (DCE), and inserted an Ag/AgCl electrode inside. When the Ag/AgCl electrode was applied to positive bias on the outer Ir/Pt coating layer, small volumes from a single cell could be injected or aspirated depending on the magnitude of voltages applied. In this case, a positive bias larger than 0.5 V results in aspiration, while <0.5 V results in injection. The femtoliter volume of liquid manipulated was calibrated with respect to applied voltages. Typically, an estimated 1,800 molecules of 3.5 kb pmaxGFP plasmid were injected into cells, and could cause cells to express green fluorescent protein (GFP) in 48 h after the injection. The transfection efficiency was evaluated to be close to 100%. With similar system, intracellular proteins could be detected. The cytoplasm could be extracted and deposited onto a coverslip. The quantification was realized by comparing the fluorescent intensity of sample vs. pure protein solutions (Shekaramiz et al., 2018).

Pourmand's group demonstrated that the double-barrel nanopipette based single cell injection, as shown in Figure 3C (reprinted from Seger et al., 2012). The double-barrel nanopipettes, as shown in Figure 3B (reprinted from Nadappuram et al., 2019), are fabricated with almost the same process with common single barrel nanopipettes, except for a glass capillary tube with a partition in the middle, in other words, with a θ shape cross section. Therefore, the requirement for an external reference electrode is not necessary by using the double-barrel nanopipette for injection. In this case, two reference electrodes insert into each barrel. The distance between the tip and cell surface could be detected by employing one barrel as part of a SICM. When close enough to the cell, the nanopipette can be precisely put in the cell cytoplasm, and then the target material can be transported by biasing one barrel against the other. With an extra barrel, the authors achieved the selective delivery of two distinct fluorescent dyes, even at varying ratios into the same single cell without cross-talk, and each single barrel was loaded with a different dye. While the voltage applied is rather high, in the range of 10–20 V, which may result in the voltage applied across the two orifices, where the resistance is very high, and thus the potential drop is significant there. In turn, it requires the entire injection process <1 min per cell on average, and up to 10 cells can be injected in <5 min (Seger et al., 2012). With similar configuration, evaluating localization of mRNA in a single cell is reported. Also, both aqueous and organic electrolyte solutions could be filled into two barrels in a nanopipette, which were used for SICM and as an electrochemical syringe, respectively. Topography with subcellular resolution, as well as the sample position, was recorded with SICM. Then, the sample was transferred to qPCR analysis to assess cellular status. They demonstrated that mRNA expression depends on cellular position, as shown in Figure 3D (reprinted from Nashimoto et al., 2016). With this double barrel nanopipette, dielectrophoretic trapping of DNA and protein was reported (Nadappuram et al., 2019). This so-called nanotweezer system is comprised of two coplanar semi-elliptical nanopipettes with a dimension of the major and minor axes about 30 nm, which are separated by a 10–20 nm thick partition inherited from the θ shape capillary. With this nanotweezer, manipulation and extraction of DNA and RNA in a single living cell were demonstrated. The authors also envisioned the possible integration of this nanotweezer with SCIM for spatial and temporal quantification of gene expression within a single cell.

Another interesting approach reported, which was named as fluid cell knife (Fluid CK), can be used as a knife to precisely cut off or heal a portion of a single cell in the original adherent culture state. The Fluid CK contains an array of four orthogonal micropipettes. In a typical process, cell lysate was released from one micropipette and drained out from two adjacent ones, forming a local laminar flow. By positioning the laminar flow close enough to a specific area of a target cell, the area covered by the lysate can be precisely “cut off” (Mao et al., 2019). In addition, local operations on target portions of a living single cell could be achieved in its adherent culture state for various types of cells, and also for temporal wound repair.

One of the most prevalent and important applications of nanopipette is single molecule detection. Among them, the most prevalent way was the resistive-pulse sensing (Wang et al., 2013). Like the nanopipette based microinjection system, the prototype device of resistive-pulse sensing is composed of two chambers via the nanopipette. Once a voltage is applied across the nanopore, ions and charged targets or analytes will be induced through the nanosized aperture, and the ion current can be monitored or recorded in this process. When the targets or analytes partially occupying the nanopipette orifice was transferred through a nanopipette, they would usually cause a conductance change and a current change pulse (Wang et al., 2013). Thus, identification of the target molecule or deduction of the interactions of the target molecules could be realized with the nanopipette based on the current-time curve to reflect the frequency, amplitude, and duration of the current pluses. In principle, there is a one-to-one correspondence between the translocation event and the current pulse. In other words, if the current pulse can be resolved in real time and turn the applied potential off at arbitrary time, the number of translocation molecule could be controlled. With this technique coming true, one can expect that a precise number of substances could be injected into or extracted from a single cell at single molecular accuracy (Bulbul et al., 2018; Nadappuram et al., 2019; Wang et al., 2019).

### Microfluidic Chip for Single-Cell Sampling

Besides methods mentioned above, some other techniques have also been used for interchanges of molecules between extracellular and intercellular environment. The adherent cells could be operated by nanopipette, however, for suspended cell, microfluidic chip is a good choice. With excellent design and processing capability, the channels in microfluidic chip allow suspended cells to be flowed, immobilized and electroporated. A multiple channel design allows parallel single cell electroporation (Khine et al., 2005). Lee, Choi and coworkers reported a nano-injection system for the delivery of biomolecules into single suspended cells (Yun et al., 2019). The system contained a hybrid (PDMS/glass) microfluidic chip, with microfluidic channel on PDMS and nanoinjection tip on solid glass. In a typical electroporation process, suspended cell was pressure driven in the microfluid channel before reaching and tightly stuck in the trapping zone, where the cell was electroporated by electric field applied through the nanoinjection tip. After electroporation, the processed and original cells were pressure driven into different channels to separated harvesting chambers. A semiquantitative dose control was accomplished by electric flied modulation of the electrokinetic pumping. In addition, the cell viability of this system is >95%, with a gene expression efficiency of up to 51%.

## Conclusion and Perspectives

In recent years, great progress has been made in the field of single cell sampling and some impressive cases have been reported. Some of the technological advances are representative and demonstrated to play powerful roles in biological and medical research. Despite their own advantages and great progress made, none of these approaches above can solve all those problems alone. Firstly, nanostaw sampling is high-throughput, and can be used to study multiple cells simultaneously, while encounter difficulties when it is used for the inspection on specific single cell, or sampling from specific sites of cells, and key factors include the diameter and density of the nanostraws in terms of the penetration validity and cell viability. Although optimality has been given in some cases, the universality has yet to be achieved on a much broader scale. Secondly, the nanopipette, AFM and FluidFM are suitable for targeting specific single cells at the cost of throughput at the same time, and the position distinctions between cytoplasm and nucleus could be easily realized with the aid of microscope. The nanopipette has little cell damage since the outer diameter of the nanopipette could be directly laser pulled down to <10 nm, while AFM or FluidFM tips at this size is on the premise of sophisticated nano-processing technology. In practice, the preparation of laser pulled nanopipette is easy and low cost. The diameter fluctuates within a certain range, and may be problematic for small diameters (<10 nm) at the cost of yield. Cell surface morphology could be mapped by AFM, FluidFM and nanopipette based SICM, yet the force feedback between the tip and the sample is an unique virtue of AFM and FluidFM. With this advantage, additional information about biomolecular interactions can be obtained at the same time of sampling.

With the rapid development of nanotechnology, one could expect the emergence of more nanomaterials and more advanced processing nano-technologies, and their applications for single cell investigation. Dimensions down to a few nanometer or even smaller, such nanomaterial would be compatible with most biomolecules on the same order of magnitude. More sophisticated functionalization with these materials would facilitate to screen the biomolecules or identify their interactions more specifically from their complex environment. The instruments developed from new principles, from upgrades of specificity of present techniques, or from the collaboration of present instruments or techniques, would provide more possibility of inspection on the biomolecules and their interactions from a more accurate temporal-spatial resolution *in-situ* and on-time.

## Author Contributions

MG and JJ conceived, designed, and supervised the manuscript. XX collected the literatures, analyzed the data, and wrote the manuscript. All authors reviewed and approved the final manuscript.

## Conflict of Interest

The authors declare that the research was conducted in the absence of any commercial or financial relationships that could be construed as a potential conflict of interest.
